# Altered metabolite accumulation in tomato fruits by coexpressing a feedback‐insensitive *AroG* and the *PhODO1 MYB‐type* transcription factor

**DOI:** 10.1111/pbi.12583

**Published:** 2016-06-22

**Authors:** Qingjun Xie, Zhongyuan Liu, Sagit Meir, Ilana Rogachev, Asaph Aharoni, Harry J. Klee, Gad Galili

**Affiliations:** ^1^ State Key Laboratory for Conservation and Utilization of Subtropical Agro‐Bioresources Guangdong Provincial Key Laboratory of Plant Molecular Breeding South China Agricultural University Guangzhou 510642 China; ^2^ Department of Plant and environmental Science Weizmann Institute of Science Rehovot 7610001 Israel; ^3^ Horticultural Sciences Department University of Florida Gainesville FL 32611‐0690 USA

**Keywords:** AroG, ODO1, tomato, fruit, secondary metabolism, shikimate pathway

## Abstract

Targeted manipulation of phenylalanine (Phe) synthesis is a potentially powerful strategy to boost biologically and economically important metabolites, including phenylpropanoids, aromatic volatiles and other specialized plant metabolites. Here, we use two transgenes to significantly increase the levels of aromatic amino acids, tomato flavour‐associated volatiles and antioxidant phenylpropanoids. Overexpression of the petunia MYB transcript factor, *ODORANT1* (*ODO1*), combined with expression of a feedback‐insensitive *E. coli* 3‐deoxy‐D‐arabino‐heptulosonate 7‐phosphate synthase (*AroG*), altered the levels of multiple primary and secondary metabolites in tomato fruit, boosting levels of multiple secondary metabolites. Our results indicate that coexpression of *AroG* and *ODO1* has a dual effect on Phe and related biosynthetic pathways: (i) positively impacting tyrosine (Tyr) and antioxidant related metabolites, including ones derived from coumaric acid and ferulic acid; (ii) negatively impacting other downstream secondary metabolites of the Phe pathway, including kaempferol‐, naringenin‐ and quercetin‐derived metabolites, as well as aromatic volatiles. The metabolite profiles were distinct from those obtained with either single transgene. In addition to providing fruits that are increased in flavour and nutritional chemicals, coexpression of the two genes provides insights into regulation of branches of phenylpropanoid metabolic pathways.

## Introduction

Plant secondary metabolites act as colour pigments, nutrients, components of flavour, protectants against ultraviolet (UV) light, signalling molecules, as well as cell wall components in plants (Liu *et al*., 2015; Maeda and Dudareva, 2012; Tzin and Galili, 2010). As the best characterized precursors for synthesis of secondary metabolites, the aromatic amino acids (AAAs), including phenylalanine (Phe), tryptophan (Trp) and tyrosine (Tyr), are derived from chorismate, the final metabolite of the shikimate pathway followed by the aromatic amino acids metabolic pathways. In fact, more than 30% of the fixed carbon in vascular plants is directed towards synthesis of aromatic amino acids via the shikimate and aromatic amino acid biosynthesis pathways (Maeda and Dudareva, 2012; Tohge *et al*., 2013; Tzin and Galili, 2010). So far, numerous genes encoding regulators of AAA biosynthesis and downstream secondary metabolic pathways have been identified in various plants species. Among them are members of the MYB family of transcription factors.

A large number of MYB regulators that participate in AAA biosynthesis have been characterized to date (Liu *et al*., 2015). For example, expression of the first gene in the shikimate pathway, 3‐deoxy‐D‐arabino‐heptulosonate 7‐phosphate synthase (DAHPS; EC 2.5.1.54), is regulated by the MYB transcription factor ATR1/MYB34 in Arabidopsis (Bender and Fink, 1998). Knockdown of *MYB8* in *Nicotiana attenuata* significantly reduces expression of all seven shikimate pathway genes, eventually leading to complete elimination of phenylpropanoid–polyamine conjugates (Kaur *et al*., 2010). The role of MYB transcription factors in controlling the Phe‐derived pathways is apparent, including in the *Solanacea* family. For example, SIMYB12 is involved in regulating biosynthesis of phenylpropanoids, particularly flavonoids, in tomato fruit (Adato *et al*., 2009; Luo *et al*., 2008). Production of the Phe‐derived phenylpropanoids/benzenoids in petunia flowers is controlled by a complex that contains a C2H2‐type zinc finger DNA‐binding protein, EPF1, and two R2R3‐type MYB transcription factors, ODORANT1 (PhODO1) and EMISSION OF BENZENOIDS II (EOBII) (Spitzer‐Rimon *et al*., 2010; Van Moerkercke *et al*., 2011; Verdonk *et al*., 2005; ). The R2R3‐MYB‐like gene, *EOBI*, has been implicated in the direct regulation of *PhODO1* (Spitzer‐Rimon *et al*., 2012). Notably, knockdown of *PhODO1* in petunia results in higher accumulation of the *EOBI* transcript, suggesting a complex feedback loop between these regulatory factors. Interestingly, ectopic expression of *PhODO1* in tomato fruits resulted in higher levels of a specific subset of phenylpropanoid compounds, but no changes were observed in the levels of Phe‐derived flavour volatiles (Dal Cin *et al*., 2011). This result suggests that, in contrast to petunia, PhODO1 does not interact with the promoters of genes responsible for Phe‐derived volatiles when overexpressed in tomato fruit.

To date, different strategies to promote the production of Phe‐derived secondary metabolites have been employed (e.g. Zhang *et al*., 2015). One important aspect of achieving higher phenylpropanoid content is identification of bottlenecks in the conversion of primary metabolites into secondary metabolites. Examples of enzymes associated with the synthesis of Phe‐derived volatiles include the aromatic L‐amino acid decarboxylases (Gutensohn *et al*., 2011; Tieman *et al*., 2006a), phenylacetaldehyde synthase (Kaminaga *et al*., 2006) and isoeugenol synthase 1 (Dexter *et al*., 2007). Our previous studies also indicated that overexpression of the bacterial *AroG* gene, encoding DAHPS in plants, alleviates a bottleneck in the conversion of primary to secondary metabolites, resulting in enhanced levels of multiple secondary metabolites, including aroma volatiles (Oliva *et al*., 2015; Tzin *et al*., 2012, 2013).

**Figure 1:**
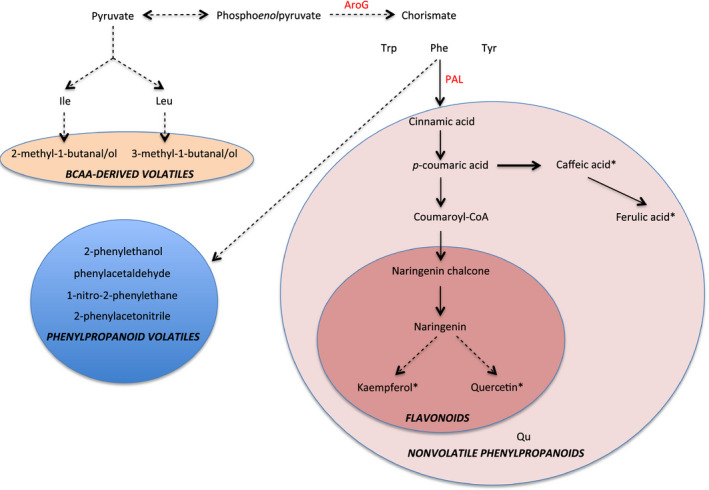
Overview of metabolic pathways altered in AroG and ODO1 expression. Metabolites with asterisks are precursors of multiple glycosylated forms.

In this study, we were interested in understanding the relationships between volatile and nonvolatile phenylpropanoid metabolites summarized in Figure 1. Specifically, our goals were as follows: (i) investigating the effects of the combination of PhODO1 and AroG in regulating the production of volatile and nonvolatile Phe‐derived secondary metabolites via metabolic engineering; (ii) producing tomato with higher flavour metabolites. Our results reveal that coexpression of *PhODO1* and *AroG* redirected the flow of phenylpropanoids to certain secondary metabolites in tomato fruit in a distinctly different way than either single transgene. While coexpression did result in lower phenylpropanoid flavour volatiles than *AroG* alone, they were still significantly higher than in wild‐type or *PhODO1* plants. Coexpressing plants rerouted Phe into multiple nonvolatile phenylpropanoids with known antioxidant properties. These results shed light on the multiple regulatory roles of ODO1 and AroG in the conversion of primary to Phe‐derived secondary metabolism. They also provide a basis for more precise metabolic engineering of tomato to produce desired target secondary metabolite products.

## Results

### Generation of tomato lines overexpressing the AroG and ODO1 proteins

**Figure 2:**
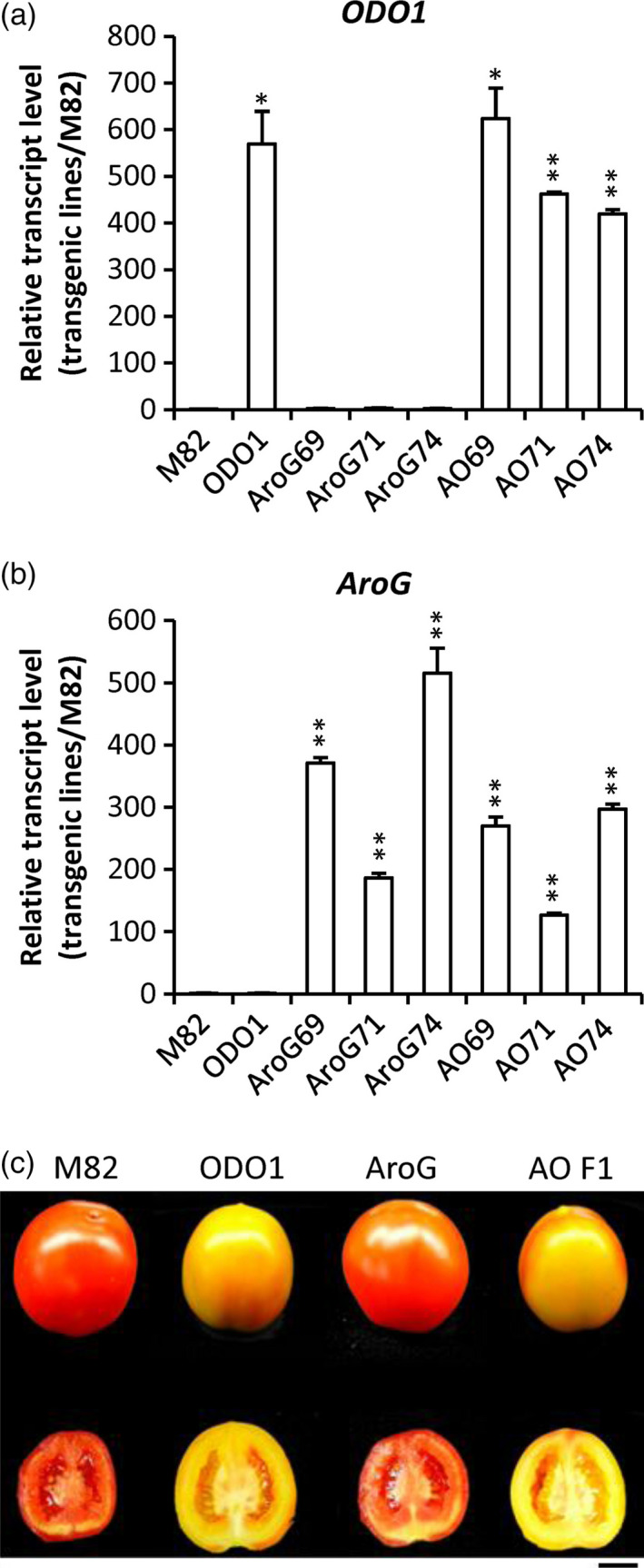
Characterization of tomato lines coexpressing *ODO1* and *AroG*. Transcript levels of *PhODO1* (a) and *AroG* (b) in fruits of the three genotypes relative to M82 (±SE). *P <* *0.05, **P < 0.01. ODO1, line 8117; AroG69/71/74, three independent lines of AroG; AO69, AroG69 × PhODO1‐8117 F1‐1; AO71, AroG71 × ODO1‐8117 F1‐9; AO74, AroG74 × ODO1‐8117 F1‐5. (c) Fruit ripening phenotypes of *AroG* and *ODO1* coexpression lines. M82, control; ODO1, line 8117 expressing *ODO1*; AroG, line expressing *AroG*; AO F1, F1 generation of lines 8117 and AroG. Bar = 1 cm.

As *AroG* and *ODO1* have each been shown to impact secondary metabolism in different ways in tomato fruit (Dal Cin *et al*., 2011; Tzin *et al*., 2013), we were interested in coexpressing these two genes to examine their effects on the levels of both volatile and nonvolatile secondary metabolites in tomato fruits (Figure 1). To this end, the *ODO1*‐overexpressing tomato line 8117 was selected for these studies as it exhibited the highest expression of all the transgenic lines (Dal Cin *et al*., 2011). Subsequently, three independent *AroG*‐overexpressing tomato lines were individually crossed with the ODO1‐8117 line to generate transgenic lines whose fruits coexpress the *AroG* and *PhODO1* transgenes (hereafter referred to as AO). Quantitative real‐time PCR (qRT‐PCR) analysis revealed that the transcript level of *PhODO1* was similar in the ODO1 transgenic plant and most of the AO lines (Figure 2a), while the expression of *AroG* was attenuated in most of the AO lines compared to their corresponding AroG parent lines (Figure 2b).

As expression of *AroG* in the AO69 (AroG69 × PhODO1) lines was only slightly reduced relative to the corresponding AroG transgenic line, we selected AO69 for subsequent studies. Consistent with previous observations (Dal Cin *et al*., 2011; Tzin *et al*., 2013), phenotypic analysis showed that overexpressing *ODO1* retarded the maturation and/or ripening of fruits while overexpression of *AroG* had no influence on ripening. Notably, the AO fruit displayed a phenotype similar to that of ODO1 transgenic line (Figure 2c).

### Metabolic changes in wild‐type, ODO1, AroG and AO fruits

**Figure 3:**
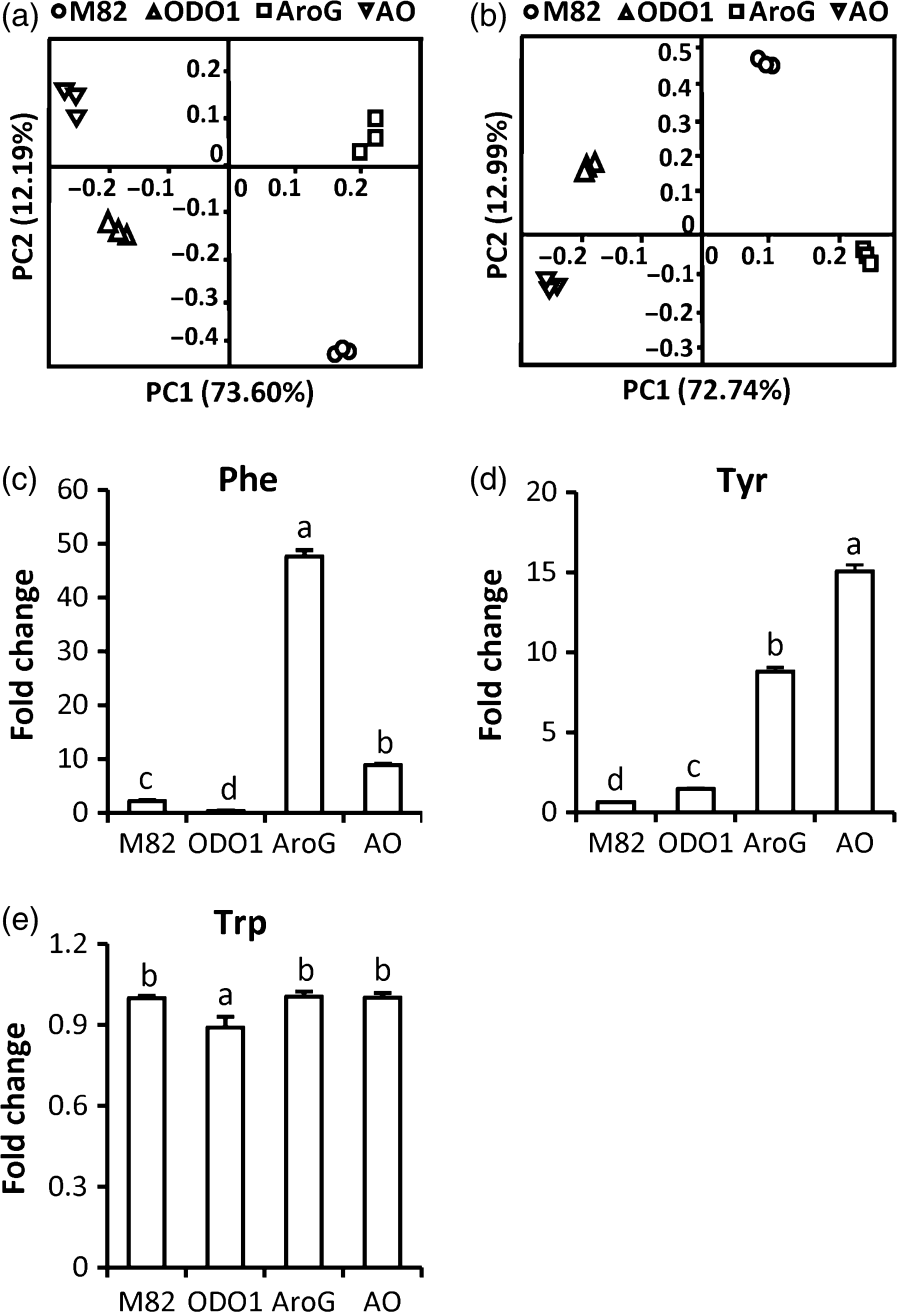
Metabolic profiles of nonvolatile phenylpropanoids in four tomato genotypes. PCA analyses of the metabolites identified in peel (a) and flesh (b) tissues. Levels of Phe (c), Tyr (d) and Trp (e) in peel and pericarp tissue of ODO1 (PhODO1‐8117), AroG (AroG69) and AO (AO69 F1‐1) lines are presented as fold change relative to M82 (*n *= 4). Letters represent significant difference among the four genotypes using ANOVA (*P *<* *0.05) followed by a Newman–Keuls test (*P *<* *0.05).

To explore the effect of the combined transgenes in AO on the metabolism of the fruit, two types of tissues (peel and flesh) from the red ripe stage were separately collected for metabolite analysis using high‐resolution liquid chromatography/mass spectrometry (LC‐MS). To obtain a general view of the differences in metabolite profiles among different genotypes, a principal component analysis (PCA) was conducted with the LC‐MS dataset. PCA of the extracts from both peel and flesh tissue showed that the metabolic profiles of wild type (cv. M82), AroG, ODO1 and AO were entirely separated from each other, indicating four distinct metabolic outcomes (Figure 3a,b). To further test whether the levels of metabolites related to the shikimate pathway were altered in AO fruit, we examined the levels of amino acids in the various genotypes. The levels of Phe were significantly reduced in ODO1, elevated in AO and very highly elevated in AroG fruits (Figure 3c). Tyr was significantly elevated in all three transgenic genotypes relative to M82 (Figure 3d). Trp levels were not significantly different from M82 in any of the transgenic genotypes (Figure 3e). Together, these results indicate that AroG has a significant positive effect on synthesis of both Phe and Tyr while minimally impacting Trp levels. ODO1, on the other hand, has an overall negative effect on Phe content while positively impacting Tyr content. Again, Trp accumulation was not impacted. The observed Phe depletion can be observed in both ODO1 fruits relative to M82 and AO plants relative to AroG. As expression of most of the genes in the shikimate and aromatic amino acid biosynthesis pathways is elevated in ODO1 fruits (Dal Cin *et al*., 2011), the overall decrease in Phe content associated with the presence of ODO1 is likely due to coordinate up‐regulation of downstream phenylpropanoid synthesis genes that substantially increase the levels of multiple phenylpropanoid compounds (described below), thus depleting the content of Phe in the fruit. Consistent with that interpretation, Tyr levels were positively impacted by the presence of the ODO1 transgene. It is interesting to note that despite the increased metabolic activity associated with the shikimate pathway, neither transgene altered Trp levels. We previously showed that *ODO1* overexpression resulted in increased transcript accumulation of chorismate mutase but not anthranilate synthase. Unexpectedly, the levels of Leu and Ile were significantly elevated in all three transgenic lines relative to M82 (Figure S1).

### Coexpression of *AroG* and *ODO1* alters the profiles of nonvolatile phenylpropanoid metabolites in fruit peel and flesh tissues

We were interested in examining the changes in the nonvolatile phenylpropanoids. Therefore, we first investigated the metabolite changes in peels of M82, AroG, ODO1 and AO fruits (Table S1). The levels of eight and 12 metabolites were significantly higher or lower in ODO1 as compared to M82, respectively (Table 1). Notably, the metabolites with lower content mainly consisted of naringenin‐derived metabolites. In AroG, there were eight significantly elevated metabolites relative to M82, including multiple coumaric acid‐derived metabolites. Only two metabolites, methylbutanol‐hexose‐pentose and phloretin‐trihexose, were significantly reduced in AroG relative to M82 (Table 1). There were substantially more metabolites altered in the AO fruit with the levels of 20 metabolites significantly increased and 11 reduced, relative to M82. Metabolites with significant increases included ferulic acid‐, kaempferol‐ and quercetin‐derived compounds. Among them, the levels of eight metabolites were significantly increased in AO as compared to those in the wild‐type, ODO1 or the AroG genotypes (Table 1). Interestingly, the pattern of metabolites with significant variations in ODO1 was similar to that of AO. For example, there were increases in ferulic acid‐ and kaempferol‐derived metabolites in ODO1 and AO as compared to M82, whereas naringenin‐ and quercetin‐derived metabolites were significantly lower in ODO1 and AO. These results suggest that ODO1 specifically alters expression of genes whose products direct biosynthesis of these secondary metabolites. In addition, a few metabolites were different from M82 in AroG and AO but not ODO1. Thus, the AO metabolite profile is a combination of the effects of both transgenes.

**Table 1:** Differentially expressed metabolites in the fruit peel of the four genotypes

Metabolite	WT	ODO1	AroG	AO
5‐Caffeoylquinic acid	1 ± 0.03 (ab)	0.57 ± 0.13 (a)	1.43 ± 0.5 (b)	0.74 ± 0.22 (ab)
Benzyl alcohol‐dihexose	1 ± 0.39 (a)	0.45 ± 0.39 (a)	5.55 ± 4.41 (b)	3.02 ± 1.2 (ab)
Benzyl alcohol‐hexose‐pentose	1 ± 0.24 (a)	0.54 ± 0.13 (a)	3.34 ± 1 (b)	3.32 ± 0.54 (b)
Caffeic acid hexose isomer 1	1 ± 0.06 (a)	0.59 ± 0.09 (a)	60.25 ± 33.12 (b)	7.07 ± 3.03 (a)
Caffeic acid hexose isomer 2	1 ± 0.03 (ab)	0.69 ± 0.21 (a)	6.74 ± 4.43 (b)	1.32 ± 0.21 (ab)
Caffeic acid hexose isomer 3	1 ± 0.09 (a)	0.47 ± 0.07 (a)	1.19 ± 0.61 (a)	**4.06 ± 0.24 (b)**
Chlorogenic acid	1 ± 0.11 (a)	0.53 ± 0.22 (a)	2.48 ± 0.93 (b)	0.86 ± 0.28 (a)
Coumaric acid hexose isomer 1	1 ± 0.71 (a)	0.4 ± 0.16 (a)	102.99 ± 72.25 (b)	59.7 ± 11.02 (ab)
Coumaric acid hexose isomer 2	1 ± 0.26 (a)	0.57 ± 0.06 (a)	8.68 ± 4.74 (b)	7.22 ± 2.38 (ab)
Coumaric acid hexose isomer 3	1 ± 0.16 (a)	22.73 ± 2.87 (a)	3.42 ± 1.48 (a)	88.03 ± 18.03 (b)
Coumaroylquinic acid	1 ± 0.12 (a)	0.69 ± 0.18 (a)	11.64 ± 5.29 (b)	3.19 ± 0.93 (a)
Dihydroxy‐methyl‐benzoic acid hexose	1 ± 0.21 (a)	3.57 ± 0.9 (b)	0.62 ± 0.06 (a)	2.93 ± 0.27 (b)
Ferulic acid hexose isomer 2	1 ± 0.12 (a)	6.35 ± 2.94 (a)	0.42 ± 0.07 (a)	**23.31 ± 9.13 (b)**
Ferulic acid hexose isomer 1	1 ± 0.56 (a)	3.72 ± 1.96 (a)	1.85 ± 0.96 (a)	**18.61 ± 3.31 (b)**
Feruloylquinic acid	1 ± 0.17 (a)	24.71 ± 6.29 (b)	1.63 ± 0.42 (a)	21.65 ± 6.04 (b)
Feruloyltyramine	1 ± 0.49 (a)	7.92 ± 2.5 (a)	3.07 ± 1.74 (a)	**21.84 ± 7.26 (b)**
Feruloylquinic acid‐O‐hexoside	1 ± 0.32 (a)	38.31 ± 12.84 (b)	3.06 ± 3.62 (a)	44.48 ± 11.63 (b)
Hydrocinnamic acid hexose	1 ± 0.25 (a)	0.26 ± 0.05 (a)	3.41 ± 1.65 (b)	4.02 ± 0.77 (b)
Hydroxybenzoic acid hexose	1 ± 0.09 (b)	0.13 ± 0.02 (a)	1.29 ± 0.28 (b)	0.29 ± 0.09 (a)
Hydroxylated naringenin (Eriodictyol)	1 ± 0.07 (b)	0.15 ± 0.05 (a)	0.7 ± 0.63 (ab)	0.28 ± 0.12 (ab)
Hydroxylated naringenin chalcone	1 ± 0.05 (b)	0.1 ± 0.01 (a)	0.56 ± 0.49 (ab)	0.07 ± 0.02 (a)
Hydroxy‐Lycoperoside A, Hydroxy‐Lycoperoside B or Hydroxy‐Lycoperoside C	1 ± 0.09 (ab)	1.13 ± 0.31 (b)	0.45 ± 0.15 (a)	0.97 ± 0.31 (ab)
Kaempferol‐hexose‐deoxyhexose‐hexose	1 ± 0.32 (a)	2.41 ± 0.29 (b)	0.98 ± 0.33 (a)	2.27 ± 0.43 (b)
Kaempferol‐3‐O‐feruloyl triglucoside‐7‐O‐glucoside	1 ± 0.71 (a)	62.06 ± 16.48 (b)	1.7 ± 0.81 (a)	63.52 ± 10.84 (b)
kaempferol‐3‐O‐p‐coumaroyl triglucoside‐7‐O‐glucoside	1 ± 0.2 (a)	55.5 ± 10.67 (b)	4.82 ± 2.74 (a)	94.11 ± 34.44 (b)
Kaempferol‐glucose‐rhamnose	1 ± 0.23 (b)	0.33 ± 0.11 (a)	0.47 ± 0.37 (ab)	0.21 ± 0.11 (a)
Kaempferol‐hexose‐deoxyhexose‐pentose	1 ± 0.1 (b)	0.28 ± 0.02 (a)	0.64 ± 0.45 (ab)	0.37 ± 0.05 (a)
Lycoperoside A/B or Lycoperoside C	1 ± 0.07 (a)	1.87 ± 0.39 (b)	0.62 ± 0.29 (a)	0.82 ± 0.37 (a)
Methylbutanol‐hexose‐pentose	1 ± 0.06 (b)	1.5 ± 0.31 (b)	0.17 ± 0.03 (a)	**2.74 ± 0.3 (c)**
Naringenin	1 ± 0.21 (b)	0.1 ± 0.02 (a)	0.62 ± 0.45 (ab)	0.08 ± 0.05 (a)
Naringenin chalcone‐dihexose	1 ± 0.5 (a)	1.16 ± 0.17 (a)	5.61 ± 3.87 (ab)	9.39 ± 1.06 (b)
Naringenin chalcone‐hexose isomer 2	1 ± 0.14 (b)	0.17 ± 0.02 (a)	0.77 ± 0.58 (ab)	0.28 ± 0.16 (ab)
Naringenin hexose or Naringenin chalcone‐hexose	1 ± 0.2 (ab)	0.29 ± 0.02 (a)	0.75 ± 0.45 (ab)	1.5 ± 0.45 (b)
Naringenin‐dihexose isomer 1	1 ± 0.24 (b)	0.26 ± 0.11 (a)	0.83 ± 0.42 (ab)	0.21 ± 0.11 (a)
Naringenin‐dihexose isomer 2	1 ± 0.12 (a)	1.61 ± 0.06 (a)	1.34 ± 0.89 (a)	**3.31 ± 0.45 (b)**
Phloretin‐di‐C‐hexose	1 ± 0.08 (b)	0.18 ± 0.05 (a)	0.68 ± 0.5 (ab)	0.19 ± 0.06 (a)
Phloretin‐trihexose	1 ± 0.08 (b)	0.11 ± 0.03 (a)	0.36 ± 0.26 (a)	0.18 ± 0.05 (a)
Quercetin‐hexose‐hexose	1 ± 0.21 (a)	0.75 ± 0.07 (a)	1.63 ± 0.82 (a)	**3.53 ± 0.97 (b)**
Quercetin‐dihexose‐deoxyhexose	1 ± 0.2 (a)	2.11 ± 0.24 (c)	1.18 ± 0.42 (ab)	2.06 ± 0.46 (bc)
Quercetin‐dihexose‐deoxyhexose‐p‐coumaric acid	1 ± 0.07 (a)	4.9 ± 0.86 (ab)	1.98 ± 0.94 (ab)	5.56 ± 2.91 (b)
Quercetin‐hexose‐deoxyhexose‐pentose	1 ± 0.03 (b)	0.24 ± 0.02 (a)	0.58 ± 0.37 (ab)	0.14 ± 0.06 (a)
Quercetin‐hexose‐deoxyhexose‐pentose‐p‐coumaric acid	1 ± 0.07 (ab)	0.1 ± 0.02 (a)	4.33 ± 2.57 (b)	0.38 ± 0.15 (a)
Quercetin‐O‐dihexose‐O‐deoxyhexose	1 ± 0.1 (b)	0.32 ± 0.02 (a)	0.71 ± 0.2 (b)	0.27 ± 0.13 (a)
Tomatine	1 ± 0.09 (ab)	1.31 ± 0.26 (b)	0.48 ± 0.17 (a)	0.74 ± 0.3 (ab)
Tricaffeoylquinic acid	1 ± 0.13 (bc)	0.1 ± 0.02 (ab)	1.09 ± 0.69 (c)	0.07 ± 0.05 (a)
Tryptophan	1 ± 0.09 (ab)	0.28 ± 0.11 (a)	1.28 ± 0.58 (b)	0.83 ± 0.06 (ab)

Numbers (n = 3; mean ± SE) are the fold change as compared to wild type (M82), and the number in bold indicate significant up‐regulation of the corresponding metabolites in AO, as compared to that in the other three genotypes. Boxes in yellow or green represent significant up‐regulation or down‐regulation of the level of metabolite in corresponding genotype as compared to that in wild type. The letters in parentheses represent significant difference among the four genotypes by using ANOVA (*P *<* *0.05) and the Tukey test for corrections for multiple comparisons (*P *<* *0.05).

A total of 40 phenylpropanoid metabolites were putatively identified in the flesh tissues of the four genotypes (Table S1). Of these metabolites, the levels of 15 were significantly different in one or more of the transgenic lines (Table 2). Our results indicate that three metabolites were elevated and one was reduced in ODO1 as compared to M82, whereas only one metabolite in AroG was significantly elevated (Table 2). However, 14 of the 15 metabolites were elevated in AO as compared to M82. These metabolites included caffeic acid‐, coumaric acid‐ and ferulate‐derived metabolites. These results indicate that combination of ODO1 and AroG significantly alters metabolite flow into Phe‐derived metabolites in tomato flesh. We conclude that coexpression of both the ODO1 and AroG transgenes promotes the accumulation of coumaric acid‐, caffeic acid‐ and ferulic acid‐derived metabolites at the expense of naringenin‐ and quercetin‐derived secondary metabolites.

**Table 2:** Differentially expressed metabolites in the flesh of AO fruits

Metabolites	WT	ODO1	AroG	AO
Caffeic acid hexose isomer 1	1 ± 0.36 (a)	0.43 ± 0.09 (a)	17.81 ± 11.61 (ab)	27.56 ± 9.07 (b)
Caffeic acid hexose isomer 2	1 ± 0.09 (a)	1.17 ± 0.68 (a)	5.24 ± 4.06 (ab)	8.63 ± 2.52 (b)
Caffeic acid hexose isomer 3	1 ± 0.09 (a)	1.78 ± 0.44 (a)	0.73 ± 0.56 (a)	**8.17 ± 0.66 (b)**
Coumaric acid hexose isomer 1	1 ± 0.3 (a)	0.87 ± 0.37 (a)	2.1 ± 1.6 (a)	**8.1 ± 1.66 (b)**
Coumaric acid hexose isomer 2	1 ± 0.14 (a)	0.99 ± 0.27 (a)	1.25 ± 0.88 (a)	**9.08 ± 2.18 (b)**
Coumaric acid hexose isomer 3	1 ± 0.54 (a)	5.56 ± 1.94 (a)	4.85 ± 3.03 (a)	**31.06 ± 7.15 (b)**
Coumaroylquinic acid	1 ± 0.3 (ab)	0.77 ± 0.18 (a)	1.24 ± 0.94 (ab)	5.28 ± 1.21 (b)
Ferulic acid hexose isomer 1	1 ± 0.07 (a)	0.81 ± 0.26 (a)	0.85 ± 0.57 (a)	**3.77 ± 2.02 (b)**
Feruloylquinic acid	1 ± 0.25 (a)	5.26 ± 0.7 (bc)	2.01 ± 1.46 (ab)	**13.41 ± 2.31 (c)**
Feruloylquinic acid‐O‐hexoside	1 ± 0.23 (a)	14.88 ± 7.7 (b)	0.83 ± 0.43 (a)	**27.66 ± 3.8 (c)**
Hydrocinnamic acid hexose	1 ± 0.25 (a)	3.77 ± 1.11 (a)	2.21 ± 1.93 (a)	**32.22 ± 2.86 (b)**
Methylbutanol‐hexose‐pentose	1 ± 0.45 (a)	10.9 ± 1.53 (b)	0.18 ± 0.15 (a)	4.23 ± 1.48 (c)
Naringenin chalcone	1 ± 0.31 (b)	0.06 ± 0.01 (a)	0.44 ± 0.32 (ab)	0.07 ± 0.04 (a)
Tyrosine	1 ± 0.1 (a)	0.82 ± 0.14 (a)	7.27 ± 3.48 (b)	**21.33 ± 2.79 (c)**

Numbers (*n *= 3; mean ± SE) are the fold change as compared to wild type (M82), and the numbers in bold indicate significant up‐regulation of the corresponding metabolites in AO, as compared to that in the other three genotypes. Boxes in yellow or green represent the up‐regulation or down‐regulation of the level of metabolite in corresponding genotype as compared to that in wild type. The letters in parentheses represent significant difference among the four genotypes by using ANOVA (*P *<* *0.05) and the Tukey test for corrections for multiple comparisons (*P *<* *0.05).

### Coexpression of AroG and ODO1 alters the profiles of multiple volatile metabolites

**Figure 4:**
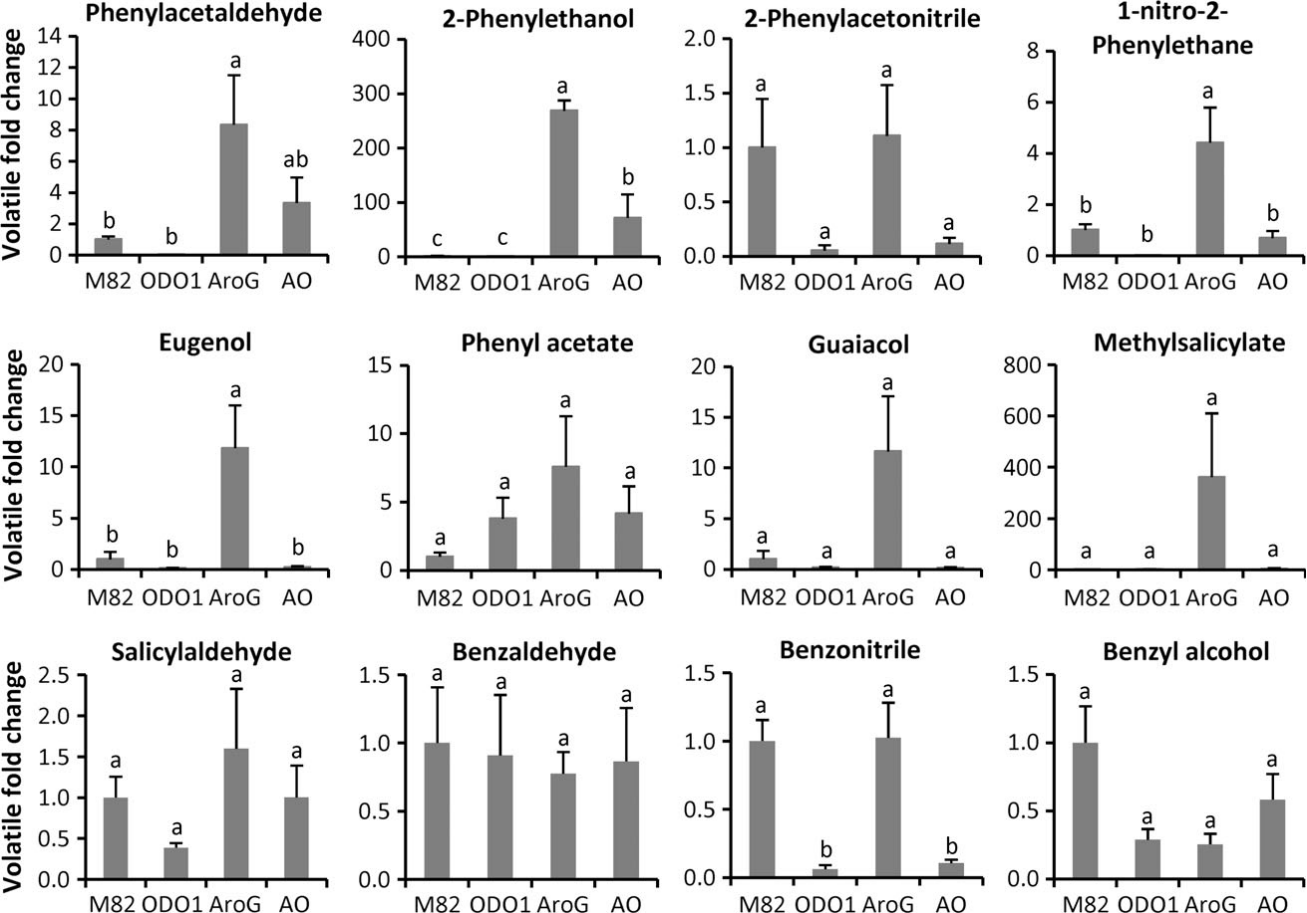
Effect of *ODO1*,* AroG* and the combination on benzenoid–phenylpropanoid volatile content. Levels (±SE) of phenylpropanoid volatiles in fruits of ODO1 (PhODO1‐8117), AroG (AroG69) and AO (AO69 F1‐1) lines. Letters represent significant difference among the four genotypes using ANOVA (*P *<* *0.05) followed by a Newman–Keuls test (*P *<* *0.05). Results are presented as fold change of each volatile relative to M82 (*n *= 3).

To further explore the effects of transgene expression, we analysed volatile compounds in M82, ODO1, AroG and AO fruits. A total of 34 compounds were examined, including several derived from Phe (Figure 4 and Table S2). PCA analysis indicated that the volatile profiles of the four genotypes were significantly separated (Figure S2). As previously reported (Dal Cin *et al*., 2011), overexpression of ODO1 did not lead to increases in phenylpropanoid volatiles, most likely because of the large increase in metabolic flux directing Phe into nonvolatile phenylpropanoids. In contrast, as might be expected from the relatively higher Phe content, AroG fruits contained significantly higher levels of several of these volatiles, including phenylacetaldehyde, 2‐phenylethanol, 1‐nitro‐2‐phenylethane and eugenol. Consistent with the greatly increased metabolic flux into nonvolatile phenylpropanoids caused by ODO1, the AO fruits contained far less phenylpropanoid volatiles than did the AroG fruits. However, one important flavour volatile, 2‐phenylethanol, was significantly higher in the AO fruits relative to M82.

**Figure 5:**
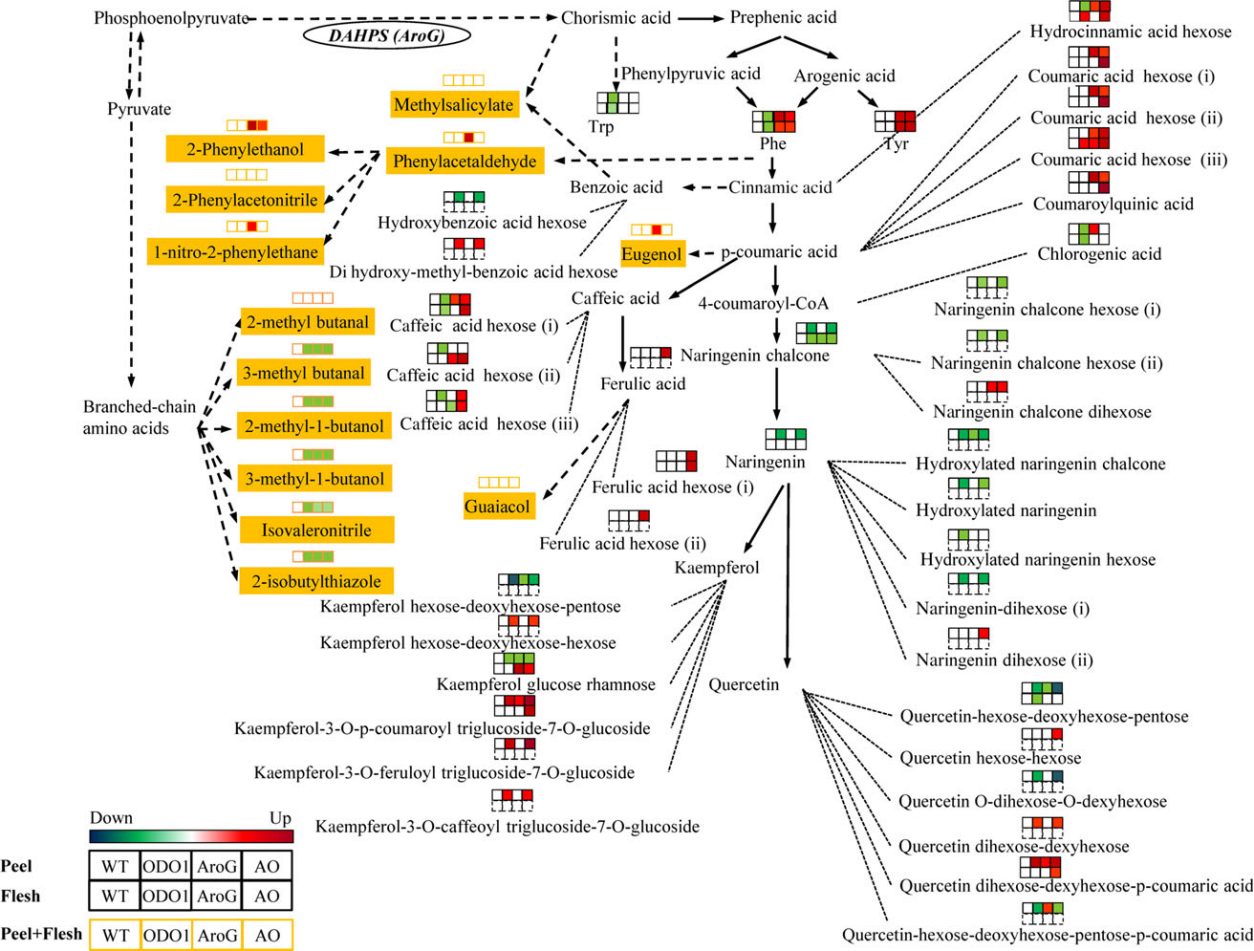
Changes of the primary and secondary metabolites in the four tomato genotypes. Green boxes indicate reduced levels, while red boxes indicate increased levels of the given metabolites. Volatile metabolites are highlighted in orange. Boxes with black dashed lines represent no corresponding metabolites detected in the flesh tissue; Boxes with orange outlines represent volatile metabolites detected from both peel and flesh tissue.

Interestingly, significantly lower levels of the branched‐chain amino acid (BCAA)‐associated volatiles were detected in all three transgenic lines relative to M82 (Figures 5 and S3). This result contrasts with the measured increases in the levels of Leu and Ile observed in these fruits (Figure S1). It should be noted that the likely precursors of the volatiles are not the amino acids but rather 2‐ketoisocaproate and 2‐keto‐3‐methylvalerate. These two molecules are both the immediate precursors and the first catabolic products of leucine and isoleucine, respectively. Thus, a larger content of free amino acids may restrict the availability of substrates for volatile synthesis.

## Discussion

Phenylalanine is the precursor of many important flavour and nutritional chemicals present in the tomato fruit. Phe‐derived volatiles that contribute to flavour include phenylacetaldehyde, 2‐phenylethanol, 1‐nitro‐2‐phenylethane and benzyl cyanide, among others (Dudareva *et al*., 2004; Tieman *et al*., 2006a). Phenylpropanoids have been associated with health for their antioxidant properties (Korkina, 2007). They are also associated with plant defence against pathogens (Korkina, 2007; Naoumkina *et al*., 2010). Thus, an understanding of the regulation of their biosynthesis is an essential step in manipulating levels of these valuable compounds in the tomato fruit.

One way to elucidate the rate‐limiting steps in metabolic pathways is to perturb the system through transgenic expression of key controlling elements and observe the effects of that perturbation on metabolite accumulation. In the case of Phe and the downstream phenylpropanoids, we have two valuable tools at our disposal. The first tool is a gene encoding a mutant *E. coli* DAHPS (Oliva *et al*., 2015; Tzin *et al*., 2012, 2013). This enzyme is the first step in the shikimate pathway that leads to synthesis of aromatic amino acids. The enzyme is normally feedback‐inhibited by Phe, but the mutant is feedback insensitive. Transgenic expression of the mutated *E. coli* enzyme in Arabidopsis (Tzin *et al*., 2012) and tomato (Tzin *et al*., 2013) plants has demonstrated that this first step in the shikimate pathway is normally a critical metabolic bottleneck that can be bypassed with the appropriate transgene. The second tool is a transgene encoding PhODO1, a petunia MYB transcription factor. Overexpression of this gene in tomato results in higher expression of genes encoding most of the steps for synthesis of Phe and Tyr as well as multiple enzymes responsible for synthesis of many nonvolatile phenylpropanoids (Dal Cin *et al*., 2011). Notably, *ODO1* overexpression in tomato causes higher expression of multiple isoforms of *PHENYLALANINE AMMONIA LYASE* (*PAL*), the first step in synthesis of most phenylpropanoids. Here, we have combined expression of the mutant *E. coli DAHPS* and *ODO1* in a single plant and observed the effects of that overexpression on a broad array of volatile and nonvolatile phenylpropanoid metabolites in tomato fruits. The overall effects on metabolite content are summarized in Figure 5.

*AroG* expression alone has a large, positive effect on Phe content (Figure 3) but a minimal effect on nonvolatile phenylpropanoids in the fruit flesh; only one of 40 measured compounds was significantly higher (Table 2). Several additional compounds were significantly increased in fruit peel (Table 1), but overall, the large increase in Phe did not have a major impact on chemicals downstream of PAL. This result suggests that *PAL* expression and not the amount of available Phe is the primary determinant of commitment to nonvolatile phenylpropanoid catabolism. In the absence of increased *PAL* expression, Phe content in AroG plants increases without a major impact on these phenylpropanoids. Some part of that increased available Phe does, however, get shunted into phenylpropanoid volatile synthesis (Figure 4). The first committed step to aromatic volatile synthesis is catalysed by a family of aromatic amino acid decarboxylases (AADC) (Tieman *et al*., 2006a). Although expression of *AADC* genes does impact volatile content, the present results indicate that availability of Phe can influence the output of this pathway.

Overexpression of *ODO1* alone actually led to a decrease in the content of Phe in the fruit. This decrease was accompanied by a major redistribution of multiple phenylpropanoids. Many exhibited significant increases, particularly those derived from ferulic acid, while others such as flavonoids were lower. We have previously reported increased fluxes of 7‐ to 100‐fold into some phenylpropanoids in *ODO1* overexpressing tomato fruits (Dal Cin *et al*., 2011) and these results are consistent with those earlier findings. That increased metabolic demand results in a reduction in the amount of free Phe and that smaller available Phe, in turn, leads to reduced levels of the phenylpropanoid volatiles (Figure 4).

The chemical contents of AO fruits exhibit properties of both *ODO1* and *AroG* expression. However, the dominant effect of *ODO1* expression is readily apparent. Fruit peel and flesh contain very high levels of nonvolatile phenylpropanoids (Tables 1 and 2). In many instances, the levels are substantially higher than those observed with either single transgene. For example, the levels of multiple caffeic acid and coumaric acid sugar conjugates are much higher in AO plant flesh. This significantly higher phenylpropanoid content is presumably due to the combination of high expression of genes encoding PAL and other downstream metabolic enzymes with much higher availability of Phe catalysed by the presence of AroG. That increased demand is evidenced by the lower level of Phe in AO relative to AroG fruit. That reduction of available Phe also impacts the ability to synthesize phenylpropanoid volatiles. Phe levels are intermediate between levels observed in M82 and AroG fruits, as are the levels of these volatiles. Indeed, only 2‐phenylethanol remains significantly higher than M82 in the AO fruits. Taken together, we conclude that the demand imposed by higher *PAL* gene expression shunts Phe into nonvolatile phenylpropanoids at the expense of the phenylpropanoid volatiles.

The level of Tyr in the transgenic lines is consistent with the above arguments. Expression of *ODO1* in fruits increases expression of most of the genes responsible for Phe and Tyr synthesis (Dal Cin *et al*., 2011). But the downstream demand for Tyr is far less than for Phe. Thus, ODO1 fruits have significantly elevated Tyr content than do M82 fruits (Figure 3). AroG, by itself, also very significantly increases Tyr content relative to M82. The combination of the two transgenes in AO is synergistic with yet again higher levels of Tyr. Thus, the reduction of Phe in AO fruits relative to AroG is likely due to increased demand for synthesis of nonvolatile phenylpropanoids.

Much attention has been paid to the health promoting activities associated with phenylpropanoids, particularly anthocyanins and flavonols in tomato fruit in particular (Bovy *et al*., 2002; Butelli *et al*., 2008; Luo *et al*., 2008). Synthesis of these compounds is regulated by MYB transcription factors [reviewed in (Liu *et al*., 2015)] and overexpression of MYBs can lead to substantially increased flavonol and anthocyanin contents (Bovy *et al*., 2002; Butelli *et al*., 2008). Overexpression of *ODO1*, also a MYB transcription factor, actually reduced the content of naringenin chalcone, a key intermediate in synthesis of anthocyanins (Table 2). The hydroxycinnamic acids, including caffeic, chlorogenic and coumaric acids, are also antioxidants and antimicrobials (Korkina, 2007; Luo *et al*., 2008; Naoumkina *et al*., 2010). Thus, fruits with higher levels of the hydroxycinnamic acids are also potentially valuable. While the ODO1 fruits have higher levels of hydroxycinnamic acids, the AO plants have significantly more of both the volatile and nonvolatile phenylpropanoids. They also contain significantly more Phe and Tyr, both essential amino acids. Thus, the AO fruits have the potential to be both more flavourful and contain higher levels of antioxidants. Whether they are perceived as having superior flavour remains to be determined. It would also be of great interest to combine AroG expression with the MYB genes that direct higher flavonol and anthocyanin synthesis such as MYB12 (Luo *et al*., 2008) and MYB112 (Lotkowska *et al*., 2015), as we would predict that these combinations would lead to further increases in those chemicals.

## Conclusion

Here, we describe plants altered in both soluble and volatile phenylpropanoid composition as a consequence of transgenic expression of a plant MYB transcription factor, a bacterial feedback‐insensitive DAHPS (AroG) and a combination of the two. Expression of either single gene or the combination of the two led to three distinct metabolic outcomes. Expression of AroG alone resulted in higher accumulation of volatile and nonvolatile phenylpropanoids. Expression of ODO1 alone resulted in large increases in nonvolatile phenylpropanoids with minimal effect on volatiles, presumably due to the large increase in *PAL* expression, leading to diversion of Phe into the nonvolatile pathways. Coexpression of both genes resulted in further increases in nonvolatile phenylpropanoids, especially the hydroxycinnamic acids, with some increase in volatile phenylpropanoids. The consequences of these manipulations indicate a way forward for increasing both flavour‐associated volatile and antioxidant phenylpropanoids.

## Materials and methods

### Plant material and growth conditions

Greenhouse‐grown plants used in this study were in the M82 background, including the *AroG* and *ODO1* transgenic plants. Each biological repeat was a mixture of five to six individual fruit in the ripe red stage (manually dissected peel and flesh tissues without the gel and seeds). To generate the *AroG* and *ODO1* coexpression lines, homozygous ODO1 lines were crossed with three independent homozygous AroG transgenic lines and the resulting F1 plants were used for this study.

### Metabolic profiling using high‐resolution LC‐MS

Nontargeted metabolic analysis was performed using 100 mg of frozen powder from tomato peel and flesh tissues, extracted in 80% methanol. Samples were analysed using an UPLC/qTOF system (HDMS Synapt; Waters Milford MA, USA), with the UPLC C18 column connected online to a photodiode array detector and then to the MS detector, in MS^E^ acquisition mode. Sample preparation and injection conditions were performed as previously described (Mintz‐Oron *et al*., 2008). Analysis of the raw LC‐MS (UPLC/qTOF‐MS) data was performed using the XCMS software from the Bioconductor package (v. 2.1) for the R statistical language (v. 2.6.1) that performs chromatogram alignment, mass signal detection and peak integration (Smith *et al*., 2006). XCMS was used with the following parameters: fwhm = 10.8, step = 0.05, steps = 4, mzdiff = 0.07, snthresh = 8, max = 1000. Injections of samples in the positive and negative ionization modes were performed in separate injection sets and preprocessing was done for each ionization mode independently. The list of putatively identified compounds (totally 69 metabolites in both peel and flesh), including their exact masses, retention times and the main fragments, is present in Supplemental Table 1. Differential mass ions were determined using a Student's *t*‐test (JMP software) and 17 metabolites whose levels were significantly different from the control M82 were subsequently identified. Principal component analysis (PCA) was performed with the T‐MEV4 software (Scholz *et al*., 2004). A Student's *t*‐test analysis was performed on metabolites using the JMP software (SAS).

### Volatiles Collection and Analysis

Collection of volatile compounds was performed as described previously (Tieman *et al*., 2006b). In brief, volatiles were collected from chopped ripe fruits (peel and flesh) during a 1‐h period. The volatiles were trapped on SuperQ resin and subsequently eluted with methylene chloride using nonyl acetate as an internal control. The samples were separated on a DB‐5 column (Agilent, www.agilent.com) and analysed on an Agilent 6890N gas chromatograph equipped with a flame ionization detector. Retention times compared with known standards and identities of volatile peaks were confirmed by gas chromatography/mass spectrometry (GC‐MS) (Agilent 5975 GC‐MS, www.agilent.com). The list of quantified volatile compounds is presented in Supplemental Table 2. The principal component analysis (PCA) plot was performed with MetaboAnalyst 3.0 web tool (Xia *et al*., 2015). For multiple comparisons, ANOVA was performed followed by a Newman–Keuls test with SAS V8 (SAS Institute Inc., Cary, NC, USA). The level of significance is indicated in each Figure.

### Amino Acid Purification, Derivatization and GC‐MS Analysis

Amino acid purification, derivatization and GC‐MS analysis were performed according to a previous study (Chen *et al*., 2010). Briefly, tomato pericarp tissue was frozen in liquid nitrogen and ground into fine powder. The powder was mixed with 2.5 mL of 10 mm HCl and 20 μL of 10 mg/mL methionine sulphone as internal standard in a mortar by grinding. Samples were placed in scintillation vials and shaken for 45 min. Sample tubes were centrifuged at 16 000 *g* for 3 min, supernatants were drawn through SCX SPE columns (Grace Davison Discovery Science) into a vacuum manifold, and amino acids were eluted with 1 mL of 1:1(v/v) 4 m NH_4_OH: methanol. One hundred μL aliquot of the analyte was transferred to a GC‐MS vial insert and derivatized by mixing with 15 μL of pyridine and 15 μL of methyl chloroformate (MCF). To separate the MCF derivatives from the reactive mixture, 300 μL of chloroform and 200 μL of sodium bicarbonate (50 mm) were added and vortexed. The bottom layer was transferred to a GC insert containing crystals of anhydrous sodium sulphate to dry the samples before they were used for GC‐MS analysis. The mass spectra of the MCF derivatized amino acids and internal standards were obtained in SIM acquisition mode.

### RNA extraction and quantitative real‐time PCR

Total RNA was prepared from fruit tissue using ISOLATE II RNA Plant Kit (Bioline, Taunton, MA, USA). Genomic DNA contamination was removed by DNase treatment (RQ1 RNase‐Free DNase Product, Promega, Madison, WI, USA). Quantitative PCR was performed with a StepOnePlus real‐time PCR system using SensiFAST^™^ SYBR Hi‐ROX One‐Step Kit (Bioline). The oligonucleotides used for quantitative real‐time PCR were as follows: AroG: forward 5′‐ATCACCCCACAA TATCTCGC‐3′ and reverse 5′‐AGCCACTTTAATCGTACCGTC‐3′; PhODO1: forward 5′‐GAAAACTCTTCATGCACCAC‐3′ and reverse 5′‐ TCAAAACCAAAGTCATTGATACC‐3′.

## Author contributions

A. A., H. K. and G. G. designed the research. Q. X. and Z. L. performed the experiments and data analysis assisted by S. M. and I. R. Q. X., Z. L., A. A., H. K. and G. G. wrote the manuscript.

## Supporting information


**Figure S1** Leu and Ile content in different genotypes.

**Figure S2** Principal component analysis of the volatile metabolites derived from the peel and flesh tissues in the four genotypes.

**Figure S3** Effects of *ODO1*,* AroG* and the combination on branched‐chain amino acid‐derived volatiles.

**Table S1** List of putative metabolites identified in the peel and flesh of the four genotypes by UPLC/qTOF‐MS at negative mode.

**Table S2** List of quantified volatile compounds in the four genotypes by Targeted‐GC (ng/gFW/h).
