# Supplementary figures and images for: Altered metabolite accumulation in tomato fruits by coexpressing a feedback‐insensitive AroG and the PhODO1 MYB‐type transcription factor
Source: Plant Biotechnol J. 2016 Jun 22;14(12):2300–9. doi: 10.1111/pbi.12583 (PMC5103220; doi:10.1111/pbi.12583)

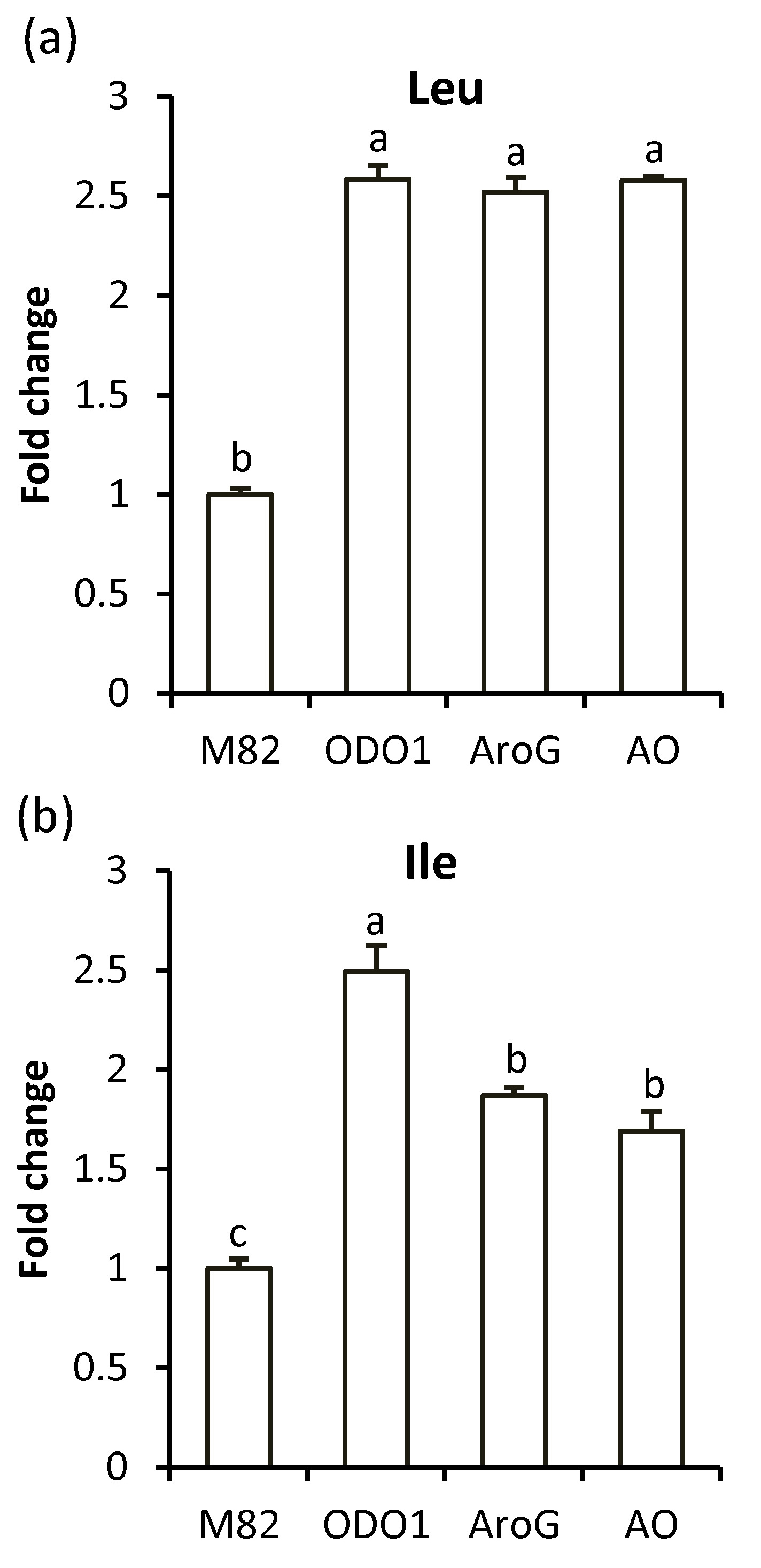

Supplement: Supplementary file 1 — Figure S1 Leu and Ile content in different genotypes. [file PBI-14-2300-s004.jpg]

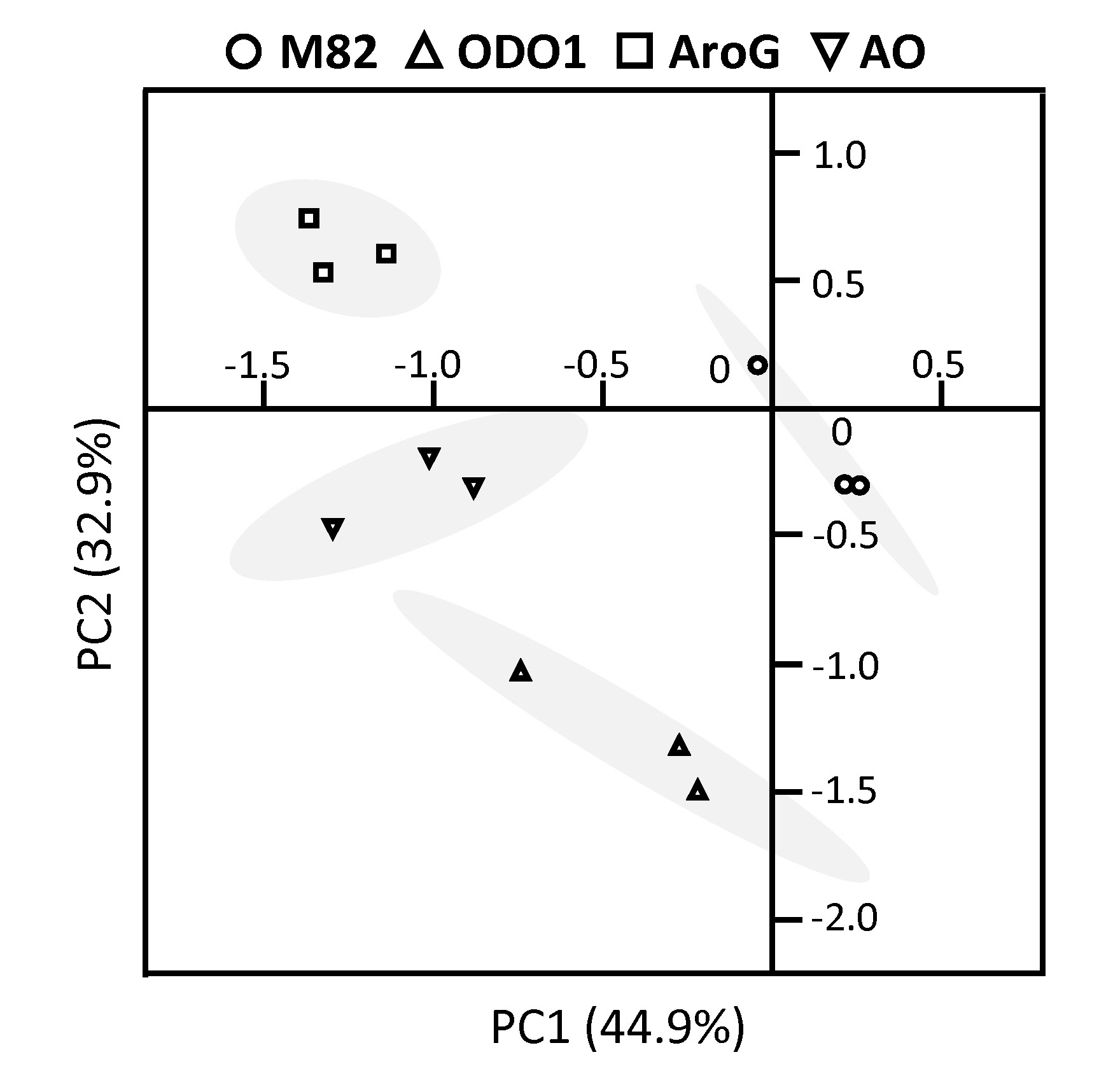

Supplement: Supplementary file 2 — Figure S2 Principal component analysis of the volatile metabolites derived from the peel and flesh tissues in the four genotypes. [file PBI-14-2300-s003.jpg]

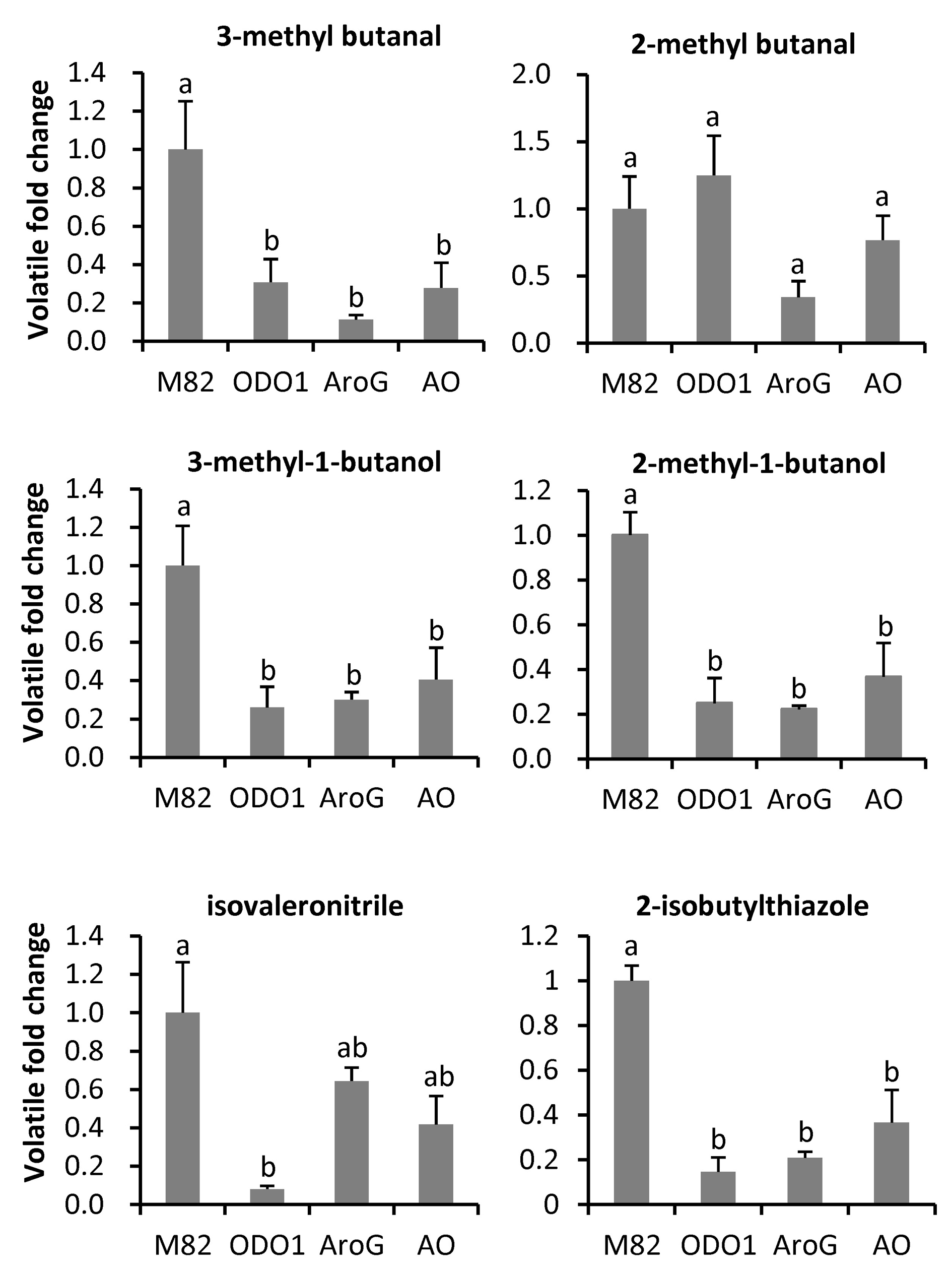

Supplement: Supplementary file 3 — Figure S3 Effects of ODO1, AroG and the combination on branched‐chain amino acid‐derived volatiles. [file PBI-14-2300-s002.jpg]
